# A learning-based algorithm for generation of synthetic participatory mapping data in 2D and 3D

**DOI:** 10.1016/j.mex.2022.101871

**Published:** 2022-09-24

**Authors:** Kamyar Hasanzadeh, Nora Fagerholm

**Affiliations:** Department of Geography and Geology, University of Turku, Turku, Finland

**Keywords:** GIS, PPGIS, Synthetic data, 3D, 2D, participatory

## Abstract

Public participation GIS (PPGIS) is a kind of spatial data that is collected through map-based surveys in which participants create map features and express their experiences and opinions associated with various places. PPGIS is widely used in urban and environmental research. PPGIS is often implemented through online surveys and points are the most common mapped features. PPGIS data provide invaluable experiential spatial knowledge. Nevertheless, collection of this data for purely methodological purposes may be costly and unnecessary. Therefore, we developed a context-aware method that can learn from previously collected PPGIS data and create a realistic dataset that can be used for methodological development purposes. The synthetic data can be generated for any desired geographical extent in both 2D and 3D, i.e. with Z coordinates. The latter is particularly important as 3D PPGIS is an emerging frontier and limited infrastructures currently exist for collection of such data. Hence, while the relevant technology is developing, spatial analytical developments can also advance using such synthetic data. This method:

• Learns from existing 2D and 3D PPGIS data in relation to the geographical context.
• Creates a realistic and context-aware simulated PPGIS point dataset.

The paper concludes by addressing the limitations and envisioning future research directions.

## Graphical abstract

**Figure:**
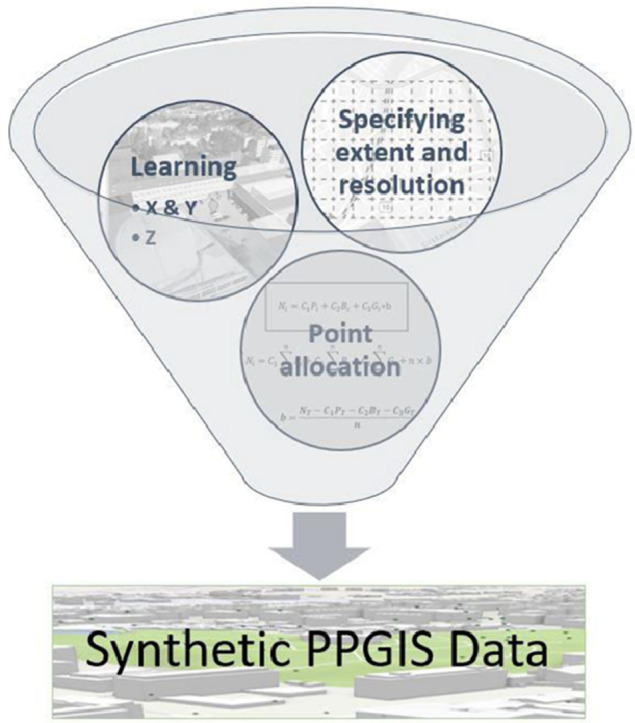

Specifications table

**Table:** 

Subject area:	Environmental Science
More specific subject area:	*Participatory mapping research (PPGIS)*
Name of your method:	*PPGIS data simulator (2D & 3D)*
Name and reference of original method:	*NA*
Resource availability:	*NA*

## Background

During the past two decades, studies have increasingly addressed people's place-based experiential knowledge in landscape and urban planning [1]. This has been predominantly addressed through participatory mapping methods (Public Participation Geographical Information Systems, PPGIS), often combining surveys with a mapping component [2]. While polylines and polygons are also commonly mapped through PPGIS surveys, points are by far the most common mapped features in these surveys. This is largely due to the fact that they are easier and more intuitive to map by the participants, and more versatile for the subsequent GIS analyses. Participatory mapping surveys are typically applied using two dimensional (2D) topographic or satellite image maps, meaning that only X and Y coordinates have been collected and Z is usually absent from the data. However, there are ongoing scholarly endeavors to make PPGIS also available in 3D.

Parallel to the dominantly empirical main body of research using PPGIS, a narrower yet significant line of research pursues methodological developments in regards to how PPGIS data is analyzed using geospatial and statistical methods (e.g., [3,4]). While such studies would certainly benefit from high quality data, a costly PPGIS survey data collection may not always be necessary as the focus of such research is primarily methodological. This is particularly relevant with new endeavors to incorporate time and altitude as extra dimensions to PPGIS studies. For such revolutions in PPGIS methods to become commonplace, further technological advancements and infrastructures are required. In the meantime, as these developments may take time, availability of high-quality synthetic data for methodological developments can be crucial.

Synthetic data refers to any data that has not been obtained by direct measurement and may be applicable to a given situation [5]. Previously, in the domain of statistics, especially population statistics [6], synthetic data were primarily viewed to be larger datasets that result from merging two or more smaller datasets [7]. However, today synthetic data can also be generated where real data is not available or for specific situations which may not be found in the original data. Synthetic data have been widely used in computational sciences and machine learning [8], and also as a means of privacy protection [9]. However, to the authors’ best of knowledge this has never been used with PPGIS methodology.

This paper takes a novel approach and develops an algorithm to create 2D and 3D synthetic PPGIS data for methodological research purposes. The algorithm is context-aware. In other words, the algorithm examines the learning data in their geographical context and aims to produce a realistic data that is comparable in its distribution and association with the physical characteristics of the given area. This is realized by including physical structural parameters adopted from previous research. These parameters are based on the previous studies exploring PPGIS and physical urban structure associations. Building density [10], population density [11], and percentage of green area are variables which we included in this algorithm [12,13].

It should be noted that PPGIS data often includes attribute data as well. In PPGIS data collection, participants are typically asked to provide additional information on each place following placing a marking (e.g., [11]). This information is linked with the spatial data as attributes describing the features. However, this study only focuses on the spatial data and hence, the attribute data is not included in the modeling.

In the following section we will describe the method in details. Subsequently, results from a test run from city of Turku in Finland will be presented to demonstrate the method's functionality and showcase the expected outcome.

## Method details

### 3D PPGIS data simulator

**Fig. 1:**
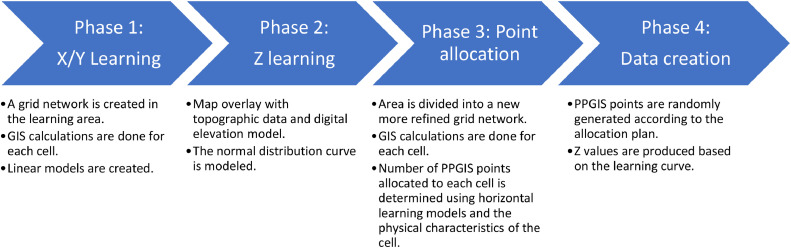
Process flow of the algorithm to create synthetic PPGIS data.

We designed a step-by-step algorithm to create the synthetic data. In summary, the algorithm goes through a set of previously collected PPGIS data and learns how the point distributions are associated with the terrain elevation and physical structure i.e., building and population density, and percentage of green area (Fig. 1: Phase 1-2). Next, what is learned in this stage is used to replicate a new dataset in the desired area roughly simulating the distribution patterns in the learning datasets (Fig. 1: Phase 3-4). A more detailed explanation of the process follows.

In the first phase the algorithm goes through a horizontal learning process. As a first step in this phase, the learning area is divided into grid cells (for example 1km x 1km). The size of cell should be chosen based on availability of computational resources, scale of contextual data available, extents of the area, and the density of learning data. For each grid cell, the percentage of green areas, building, and population density are calculated. Subsequently, the association between number of learning points in each cell and these variables are calculated using a multiple linear regression model.

The second phase involves vertical learning. As a first step in this phase, the 3D learning data is overlain with the digital elevation model (DEM) and the respective absolute elevation is recorded for each point. Next, the 3D learning data is overlain with the topographical data. If a point is located in a building, the relative position of the point compared to the building height will be measured. Finally, the vertical distribution of learning data will be captured statistically in terms of mean and standard deviation values.

In the third phase, the algorithm plans the synthetic data placement using what it has learned from the previous two phases. As a first step in this phase, the desired area is divided into smaller grid cells of for example 200m x 200m. The cell dimensions should be determined based on computational resources, extents of area, number of points needed, scale of contextual data available, and the desired output resolution. The geographical extent of output data does not have to be the same as the learning data as long as the context is reasonably consistent with that of the learning area. Inaccessible areas, such as large bodies of water, are excluded from the grid. Next, the three physical structural variables of building density, population density, and percentage of green area are calculated for each cell. Next, the number of points to be created in each cell is calculated based on the following equation:

$$N_{i}=C_{p}P_{i}+C_{b}B_{i}+C_{g}G_{i}+b$$

Where N is the number of points, P population density, B building density, G green percentage, each for the given cell i. The C values are the regression coefficients for each of these variables from the learning phase. The constant b can be calculated using the summation of the same equation for all grid cells as follows:

$$\sum_{i=1}^{n}N_{i}=C_{p}\sum_{i=1}^{n}P_{i}+C_{b}\sum_{i=1}^{n}B_{i}+C_{g}\sum_{i=1}^{n}G_{i}+n\times b\;\Rightarrow$$

$$b=\frac{N_{T}-C_{P}P_{T}-C_{B}B_{T}-C_{G}G_{T}}{n}$$

**Fig. 2:**
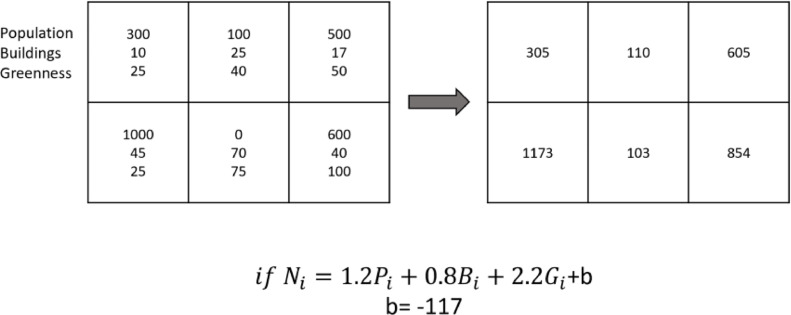
An example of calculating number of points for a six-cell grid. The coefficient values are arbitrarily chosen for the purpose of demonstration.

Where P_T_ is the total population, B_T_ is the total amount of buildings, and G_T_ is the total amount of green areas for the area, and n is the total number of grid cells. Fig. 2 illustrates an example of how number of points are calculated for each grid cell.

In the fourth phase, synthetic data is created. Number of points in each cell are determined using the equations described above and a sigmoid normalization. Each point is randomly generated within the cell in 2D. The 2D point layer is subsequently overlain with the topographic data and DEM to determine the type of location. Depending on whether the place is a building or not, the altitude is randomly estimated approximately replicating the standard deviation and average values from the vertical learning phase. When located in a building, the building height is taken into consideration in Z allocation so that the point's altitude is within the building. Optionally, some noise is added at the end to the altitude to introduce more variation to the values and simulate the imprecision and inaccuracy of user generated data.

## Algorithm's functionality and output: 3D synthetic PPGIS data for city of Turku

**Fig. 3:**
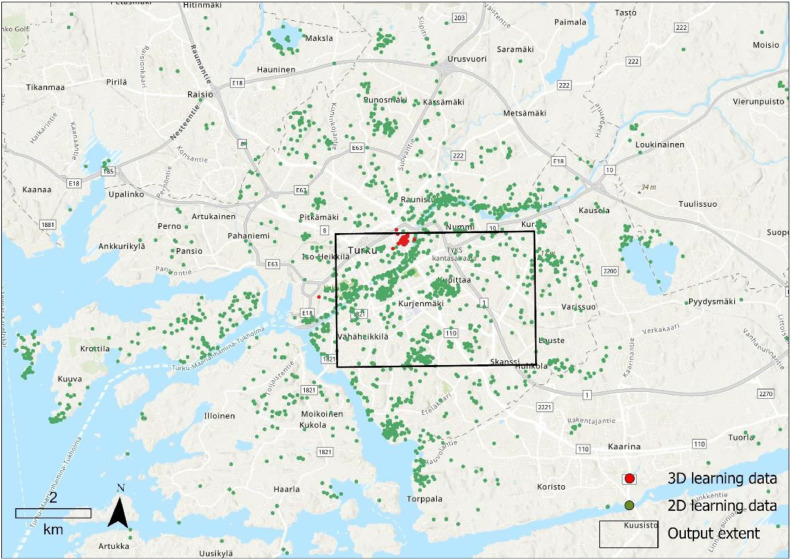
Learning data from city of Turku and the output extent.

The algorithm was implemented in Python using a number of open source libraries including Geopandas (pseudocode provided as appendix). The learning data consisted of 758 points with X, Y, and Z coordinates and 2270 2D points with only X and Y coordinates. The two datasets were both collected from city of Turku in Finland but in different occasions and as part of different projects (Fig. 3). Building data was obtained from the topographic data provided by the national land survey of Finland 1. Population data was available in 1km x 1km grids by Statistics Finland^2^. Land use data were obtained from the European CORINE Land Cover data^3^.

**Fig. 4:**
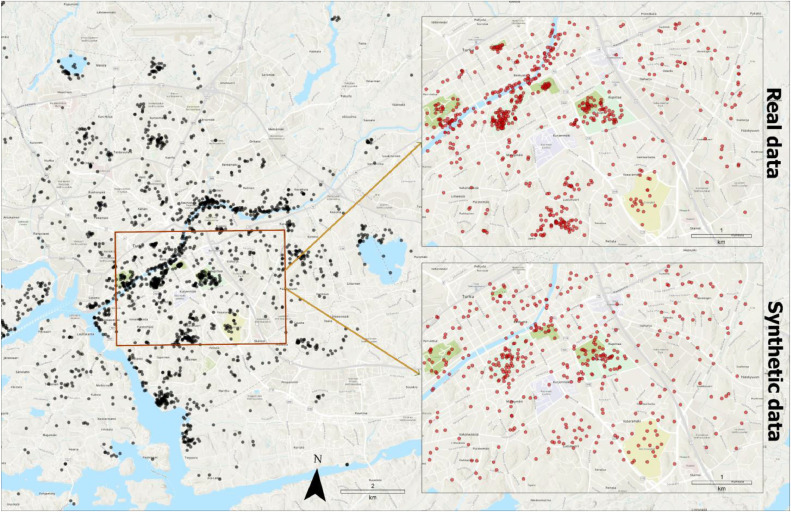
A comparison of the synthetic versus real PPGIS data in the same area.

The output extent was set to a rectangle area with the total area of approximately 18.7 km^2^ in Turku city center. The extent was divided into grid cells of 150×150 m. A total of 500 points were requested as the output, 447 of which were successfully generated. The small difference is due to the rounding effect as the algorithm rounds down the number of points allocated to each cell to the closest integer. Fig. 4 illustrates a comparison of the output synthetic data and learning data for the same area.

As expected, the clusters around green areas, densely populated areas, and areas with high building density reappear in the synthetic data. However, clusters of points around the river or other attractive geographical features are not reproduced simply because these factors were not included in the model.

## Limitations and ideas for further improvement

The method described in this paper follows a simple and intuitive approach to deliver what it is intended for, which is to generate a synthetic point dataset based on a set of learning data. For doing so, the algorithm learns from the input data by examining its distribution in relation to the population density, building density, and the amount of green areas in the geographical context. While this list of contextual variables may be enough for generating realistic data in some urban contexts, in other urban areas where other strong factors may be in force this may not be sufficient. This could be seen in the example provided above as the presence of water bodies, a river in this case, is an important factor that may influence the distribution of certain types of place-based experiential data marked by participants. Therefore, future research using this method is encouraged to investigate the main geographical characteristics of the study area and determine which contextual variables need to be added to the model for a better synthetic data generation. In addition, PPGIS data frequently includes attribute data that describes the mapped places. However, our method did not attempt to generate synthetic attribute data for the points. Hence, this requires further exploration and research in future.

Another limitation of this method is the use of multiple linear regression for the learning process. While this has the benefit of simplicity, it may not capture the spatial autocorrelation that is typical in PPGIS data [14]. While the use of high-resolution grid network can ameliorate the effect, this is something that needs to be taken into consideration specially when synthetic attribute data are also generated. Future research is encouraged to fill this gap and also consider use of other modeling techniques such as a spatial autoregressive model to tackle potential issues with spatial autocorrelation.

Another limitation of this method is in how the altitudes are modeled and generated. The algorithm described in this study assumes a normal distribution which may not be always realistic. The main reason why this was used is due to 3D learning data that was available. The 3D data used in this study was only available from one city block and, hence, was lacking the required size and variation to create a more realistic distribution model. Future research is encouraged to investigate the distribution of Z values in the learning phase and choose a model that would best capture it.

## Conclusions

We designed and implemented a method for creating synthetic PPGIS in 2D and 3D in urban areas. The goal was to promote and facilitate methodological PPGIS studies in occasions where a particular type of data is not available or feasible to collect. This is particularly relevant to the newly emerging 3D PPGIS as limited resources currently exist to facilitate collection of such data. This can facilitate methodological advancements for the analysis of PPGIS data and help reduce the technical, geospatial knowledge gap in multidisciplinary research areas applying participatory mapping for data collection.

The algorithm described in this study consists of four phases. The first two phases involve a learning process during which the algorithm learns from a set of 2D and/or 3D PPGIS data. The learning involves the horizontal and vertical distribution of map features in relation to a number of contextual variables (building and population density, and percentage of green area). This enables a context-aware creation of synthetic PPGIS data making it possible to create more realistic data which can be tailor-made for a specific situation, area or other methodological requirements. The remaining phases in the algorithm involve the creation of the data according to the learning models.

The algorithm described in this study was implemented in Python, a pseudocode version of which is provided as supplement. The method was applied in a test area in city of Turku in Finland, results of which were presented in this paper.

Future research is encouraged to improve the algorithm. The algorithm can specially benefit from a more comprehensive learning procedure. This can be for example pursued by including additional contextual factors in the learning process and using more advanced models to more accurately capture and replicate the learning data characteristics. Future research is also encouraged to examine the prospects of using similar learning-based algorithms for predictive purposes. That is to develop models which can predict the outcome of an intervention or a change in physical structure on the inhabitants using previous experiential knowledge from PPGIS [3].

## CRediT authorship contribution statement

**Kamyar Hasanzadeh:** Conceptualization, Formal analysis, Methodology, Software, Writing – original draft, Visualization. **Nora Fagerholm:** Conceptualization, Writing – review & editing, Project administration, Funding acquisition, Data curation.

## Declaration of Competing Interest

The authors declare that they have no known competing financial interests or personal relationships that could have appeared to influence the work reported in this paper.

## Appendix

#This program generates synthetic PPGIS in 2D and 3D inputs: output extents, output resolution, learning data, population data, landuse data, building data, elevation model, need3D (Boolean) function formGrid(output extents, output resolution, landuse data):

form grids based on the extent and resolution
exclude inaccessible areas such as water
return GridLearning and GridOutput

function learn2D (learning data, population data, landuse data):

do spatial join between learning data and contextual data
learn associations and form the linear function1

function allocatePoints (GridOutput, population data, landuse data, building data, elevation model):

measure contextual variables for each cell
determine the estimated point count for each cell using function1

generate random 2D points for each cell according to estimated counts if need3D is “True”:

function learn3D (learning data, elevation model, building data):
read the ground elevation at each earning data point
measure the height from ground for each point
do a spatial join and determine whether the point is located in a building or in an unbuilt area
if “building”:
estimate what floor it is located on
learn vertical distributions
assign a Z to each point so that the overall Z distribution matches learning data's Z distribution
Add random noise to Z values [optional]

Export the output data as shapefile

## Footnotes

1 https://www.maanmittauslaitos.fi/en/maps-and-spatial-data/expert-users/product-descriptions/topographic-database.

2 https://www.stat.fi/org/avoindata/paikkatietoaineistot/vaestoruutuaineisto_1km_en.html.

3 https://land.copernicus.eu/pan-european/corine-land-cover.
